# Machine learning for identifying Randomized Controlled Trials: An evaluation and practitioner's guide

**DOI:** 10.1002/jrsm.1287

**Published:** 2018-02-07

**Authors:** Iain J. Marshall, Anna Noel‐Storr, Joël Kuiper, James Thomas, Byron C. Wallace

**Affiliations:** ^1^ King's College London London UK; ^2^ University of Oxford Oxford UK; ^3^ Doctor Evidence Santa Monica CA USA; ^4^ UCL London UK; ^5^ Northeastern University Boston MA USA

## Abstract

Machine learning (ML) algorithms have proven highly accurate for identifying Randomized Controlled Trials (RCTs) but are not used much in practice, in part because the best way to make use of the technology in a typical workflow is unclear. In this work, we evaluate ML models for RCT classification (support vector machines, convolutional neural networks, and ensemble approaches). We trained and optimized support vector machine and convolutional neural network models on the titles and abstracts of the Cochrane Crowd RCT set. We evaluated the models on an external dataset (Clinical Hedges), allowing direct comparison with traditional database search filters. We estimated area under receiver operating characteristics (AUROC) using the Clinical Hedges dataset.

We demonstrate that ML approaches better discriminate between RCTs and non‐RCTs than widely used traditional database search filters at all sensitivity levels; our best‐performing model also achieved the best results to date for ML in this task (AUROC 0.987, 95% CI, 0.984‐0.989). We provide practical guidance on the role of ML in (1) systematic reviews (high‐sensitivity strategies) and (2) rapid reviews and clinical question answering (high‐precision strategies) together with recommended probability cutoffs for each use case. Finally, we provide open‐source software to enable these approaches to be used in practice.

## 1 INTRODUCTION

Randomized Controlled Trials (RCTs) are regarded as the gold standard of evidence on of the effectiveness of health interventions.^1^ Yet these articles are a small minority of the available medical literature. As of 2016, PubMed contained 26.6 million articles, of which 423 000 (1.6%) were labeled as being RCTs. *
Search conducted of PubMed (https://www.ncbi.nlm.nih.gov/pubmed) on December 2, 2016. A key task in systematic reviews in health care (and in evidence‐based medicine more widely) is identifying RCTs from large research databases. Current standard practice for identifying RCTs involves using an established *database filter*, which can automatically exclude a large proportion of non‐RCT articles; the remaining articles are being manually screened.^2^ Database filters contain combinations of text strings and database tags and have been developed by information specialists who combine search terms (see example, Box 1). †
These filters are often derived from statistical analyses of term counts in relevant and irrelevant articles, which may be iteratively combined to find an optimal sensitivity/specificity balance; in some cases, they are based on the opinion of expert librarians.

Box 1 **Cochrane Highly Sensitive Search Strategy, 2008 PubMed version^2^**

#1 randomized controlled trial [pt]

#2 controlled clinical trial [pt]

#3 randomized [tiab]

#4 placebo [tiab]

#5 drug therapy [sh]

#5 randomly [tiab]

#6 trial [tiab]

#7 groups [tiab]

#1 OR #2 OR #3 OR #4 OR #5 OR #6 OR #7 OR #8

# 10 animals [mh] NOT humans [mh]

#9 NOT #10

Automated text classification has been widely studied in natural language processing (NLP) and machine learning (ML).^3^, ^4^ In the past, discriminative linear models induced over sparse, *bag of words* (BoW) ‡
This scheme represents a text as long, sparse vector in which each element corresponds to the presence or absence of a word in the vocabulary. In the simplest case, a text is described by a sequence of 1s and 0s determined by whether each unique word in the overall corpus is present in the document of interest or not. document representations had been the prevailing approach. For example, linear‐kernel support vector machines (SVMs) were until recently considered state‐of‐the‐art text classifiers.^3^ In the past few years, neural models have come to dominate NLP generally and text classification specifically.^5^ Convolutional neural networks (CNNs), originally used for image classification, in particular have emerged as state‐of‐the‐art models for text categorization: evaluations on a range of datasets have found improvements in performance compared with linear SVM‐based approaches.^6^, ^7^ In their 2015 evaluation, Cohen and colleagues reported that an SVM‐based classifier achieved very high accuracy for RCT classification.^8^ However, the role of ML in systematic review workflows is still ill‐defined, and how ML compares with current standard practice (ie, the use of database filters) is unclear.

### 1.1. Aims

This paper seeks to evaluate ML as a strategy for identifying RCTs from health research databases. We aim to evaluate ML approaches (CNNs, SVMs, and ensembles §
The process of combining multiple models with the aim of increasing performance over any individual model.) of these approaches for RCT identification on an external gold standard dataset (the Clinical Hedges set). This allows direct comparison of ML against standard database filters. We then provide practical guidance on how ML models might be used in practice in real‐world scenarios, specifically (1) systematic reviews (ie, high‐sensitivity search) and for (2) rapid reviews or clinical question answering (high‐precision search). Finally, we present open‐source software, which implements these methods to facilitate their use in practice.

### 1.2. Use cases for RCT identification

Here, we describe 2 common use cases for an ML RCT classifier, and the performance requirements for each. Although defining discrete cutoffs in this way is somewhat arbitrary, they illustrate how ML compares with traditional database filters in common use cases. Note that one advantage of automated approaches is the ability to select a threshold suitable for a given search application; this is not possible using conventional database filters.

#### 1.2.1. Systematic review/high‐sensitivity search

The current best‐performing filters achieve near perfect sensitivity (an essential quality considering the goal of most systematic review is to retrieve *all* RCTs evaluating an intervention), but at the cost of comparatively low specificity. For this use case, we have chosen the Cochrane Highly Sensitive Search Strategy RCT filter (hereafter referred to as the Cochrane HSSS) as a comparator.^2^ The Cochrane HSSS has been found to have 98.4% sensitivity^9^ and is the standard approach for retrieving RCTs from MEDLINE currently used across the Cochrane collaboration.

**Figure 1:**
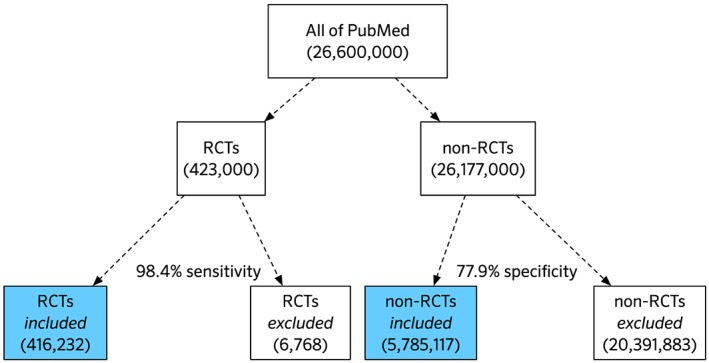
Tree diagram: The false positive burden associated with using a high‐sensitivity search compounded by RCTs being a minority class. Illustrative figures, assuming that 1.6% of all articles are RCTs (based on PubMed search; approximately 423 000 in total), and a search filter with 98.4% sensitivity and 77.9% specificity (the performance of the Cochrane HSSS based on data from McKibbon et al^9^). The 2 blue shaded boxes together represent the search retrieval. The search filter thus retrieves a total of 6 201 349 articles, of which only 416 232 (or 6.7%) are actually RCTs (being the *precision* statistic) [Colour figure can be viewed at wileyonlinelibrary.com]

Given that reports of RCTs constitute a small minority of all article types, specificity contributes substantially more to the absolute number of errors. Figure 1 shows that these performance metrics lead to a very high number of false positives in practice when searching MEDLINE. This phenomenon occurs in all search filters and ML approaches aiming to detect a minority class. Perhaps counter‐intuitively, a filter with 77.9% specificity may produce output in which only 7% of retrieved articles are genuine RCTs (see Figure 1). An effective ML strategy would maintain the 98.4% sensitivity of the Cochrane HSSS but achieve higher specificity than conventional database filters, thus reducing the burden imposed by false positives.

#### 1.2.2. Rapid review search/clinical question answering

Systematic reviews aim to be comprehensive but are time consuming to produce; it takes a median of 2 years to complete a Cochrane review from start to finish.^10^ *Rapid reviewing* describes a review in which the very strict methodological rigor of a systematic review is relaxed in exchange for faster turnaround. An appealing search filter for a rapid review, therefore, would accept modestly lower sensitivity with the trade‐off of (much) improved specificity. To act as the comparator in this scenario, we used the PubMed *publication‐type* (PT) tag used as a single search term (93.7% sensitivity, 97.6% specificity).^9^ The PT tag is manually applied by PubMed staff, and fewer than half of retrieved articles are not RCTs (56.4% precision^9^). A high‐precision strategy such as this is also well suited for real‐time question answering by clinicians, who are unlikely to have the time to manually assess large numbers of erroneously retrieved articles. Since it is a manual process, there is currently a lag time of around 250 days from publication until the PT tag is applied, although this is likely to reduce substantially in future as the original article publishers are able to supply their own PT labels. ¶
Personal communication, Dina Demner Fushman, National Library of Medicine The sensitivity of the PT tag improves gradually with time from article publication, since some retrospective correction occurs; including adding the PT tag to the missed RCTs based on data fed back by the Cochrane Collaboration.^11^ The PT tag forms a core part of all the best‐performing traditional search filters; strategies that rely heavily on this information may therefore miss recent articles that have not yet been indexed. The evaluation described above by Cohen and colleagues used the PubMed PT tag as a gold standard for training and evaluation.^8^ Although the PT tag has imperfect accuracy, it has been applied manually and at scale. Indeed, via a manual screen of apparent false positive *errors*, Cohen's team were able to show that their model had actually identified a number RCTs incorrectly tagged by PubMed.

## 2 MATERIALS AND METHODS

### 2.1 Datasets

#### 2.1.1 Clinical Hedges

The Clinical Hedges team has described their dataset in detail previously.^12^ In brief, this dataset comprises 49 028 articles from 170 journals including the full year 2000, and part of 2001. Each article was reviewed in full text by a research assistant and classified by article type. The Hedges team conducted a systematic review to identify boolean database search strategies, which were then evaluated on the Clinical Hedges set. For the purposes of the evaluation, we use the same definition of RCT as used in the Hedges team review. #
Being articles that met all 3 of the criteria: (1) original articles (reviews were excluded), (2) assessing the effects of a treatment/intervention, and (3) meeting criteria for rigor (specifically random allocation of participants to arms, 80% of participants randomized included in at least one outcome analysis, and analysis being performed consistently with study design). Use of this dataset thus allows a direct comparison with the performance of search strategies that are in widespread current use. The key strength of the Clinical Hedges dataset is that experienced researchers manually annotated full text papers. We therefore use these data as our *gold standard* for evaluation.

**Figure 2:**
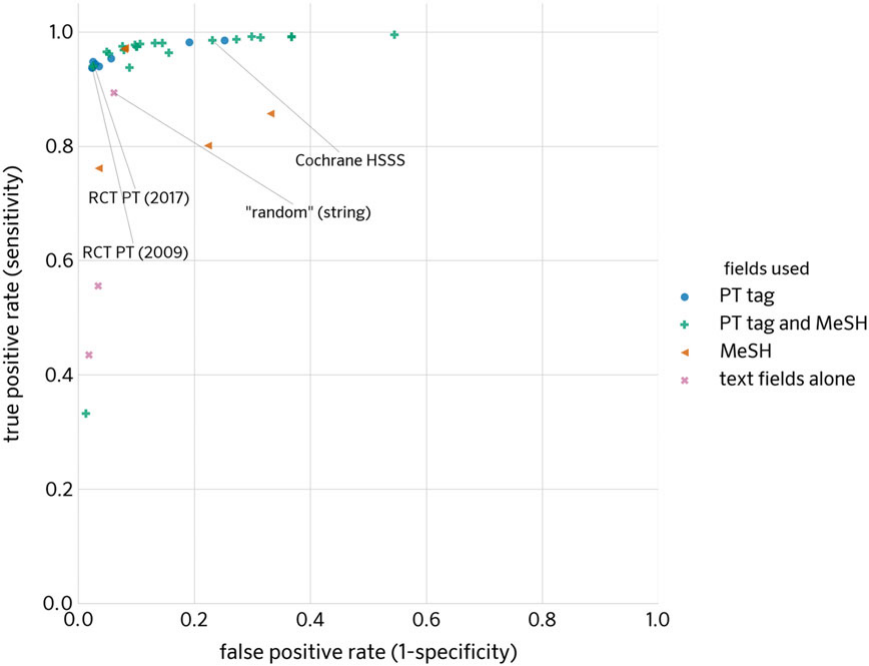
Receiver operating characteristic scatterplot for conventional database filters (based on data published by McKibbon et al,^9^ with the 2 comparator strategies from this analysis labeled. RCT PT tag, the single‐term strategy based on the manually applied *PT* tag (the high‐precision comparator); Cochrane HSSS, the Cochrane Highly Sensitive Search Strategy (the high‐sensitivity comparator) [Colour figure can be viewed at wileyonlinelibrary.com]

The Clinical Hedges team supplied a list of PubMed identifiers together with their classifications for this analysis; we excluded 3 articles that were no longer accessible in PubMed, leaving a total of 49 025 articles for this analysis. Since this same dataset has been used by the Clinical Hedges team for systematic evaluation of available database filters (see Figure 2), we may therefore directly compare ML performance against these filters.

#### 2.1.2 Cochrane crowd dataset

**Figure 3:**
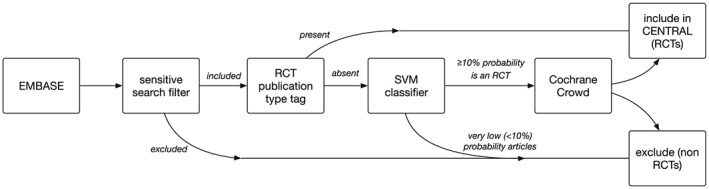
The Cochrane Crowd/EMBASE project pipeline. Source articles (titles and abstracts) are identified via a sensitive database search filter. Articles already tagged as being RCTs (via Emtree PT tag) are sent directly to CENTRAL. Articles predicted to have <10% probability of being RCTs via an SVM classifier are directly excluded. The remaining articles are considered by the crowd

The Cochrane Collaboration maintains the Cochrane Central Register of Controlled Trials (CENTRAL), which aims to include complete citation records for *all* controlled trials; this database is populated both from research databases (MEDLINE and EMBASE) and other published and unpublished sources.^13^ The current pipeline for identifying RCTs from EMBASE is a hybrid system, which incorporates a sensitive search filter, an ML classifier (SVM), and finally, a crowd of volunteer annotators (see Figure 3). The Cochrane Crowd may be considered a dataset of difficult to classify articles, since obvious RCTs and obvious non‐RCTs are removed in advance through this process.

The Cochrane Crowd project was established with the aim of improving the identification of RCTs from EMBASE. As with other databases, the proportion of RCTs in EMBASE compared with other records is very low, and in sensitive search, only approximately 4% of records are RCTs. Bibliographic records are sent to the *Cochrane Crowd* crowdsourcing platform (http://crowd.cochrane.org/index.html) where approximately 4000 volunteers inspect titles and abstracts, and categorize citations as describing an RCT or not. As of October 2016, nearly 1 million individual classifications have led, through a crowd consensus algorithm, to the identification of 22 000 RCTs out of approximately 280 000 records screened. An evaluation of the Cochrane Crowd output against double assessment by experienced researchers found that sensitivity and specificity exceeded 99%.^14^

Since January 2016, an ML algorithm has been added to identify and discard citations, which were most likely to be irrelevant. An SVM classifier was built, and citations that were predicted to have <10% probability of being an RCT are discarded. An internal evaluation found that the classifier enable 40% of citations to be discarded from the set sent to the crowd while maintaining 99.9% sensitivity.

### 2.2 Machine learning algorithms

#### 2.2.1 Linear kernel support vector machine

**Figure 4:**
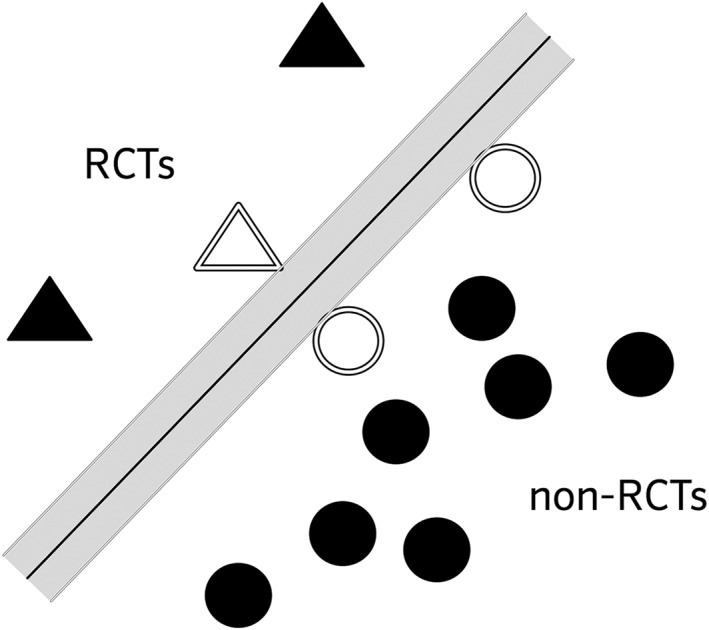
Schematic illustrating separating plane in support vector machines, here depicted in 2 dimensions. Here, the separating plane (a straight line in this two‐dimensional case) is depicted as the black line and the *margin* are depicted in gray. The instances nearest to the margin (*support vectors*) are highlighted in white

Support vector machines aim to identify a plane that separates examples from the respective classes in some (typically high‐dimensional) feature space. An infinite set of such separating planes may exist; SVMs favor those that induce the largest margin between examples from the 2 classes. This intuition is depicted schematically in Figure 4.

For text classification using linear models, one typically encodes the pieces of text to be classified (here, abstracts) via the aforementioned BoW encoding. In this scheme, an abstract is represented as a (very) long, sparse vector in which each index corresponds to a particular word (*unigram*) or pair of adjacent words (*bigram*) and is nonzero only if said unigram or bigram appears in the abstract. A *linear kernel* SVM then aims to identify a hyperplane in this high‐dimensional space that separates texts belonging to the respective categories (here, RCTs versus non‐RCTs).

#### 2.2.2 Convolutional neural network

*Neural models* have recently been shown to outperform alternative statistical models for many NLP tasks, including text classification.^5^ CNNs, in particular, have achieved state‐of‐the‐art results for text classification generally,^6^, ^7^ and for biomedical text classification tasks in particular.^15^ In place of BoW encodings, CNNs use (relatively) low‐dimensional, continuous vectors to represent words, ie, *word embeddings*, which may be induced using large amounts of *unlabeled* data.^16^, ^17^ Here, we use a set of embeddings that were induced using all abstracts indexed by PubMed.^18^

**Figure 5:**
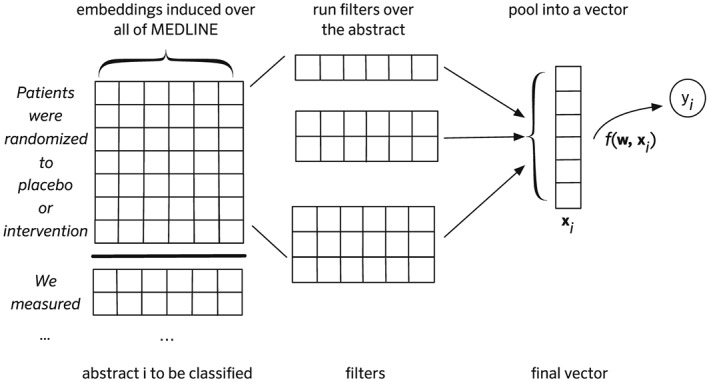
Schematic illustrating convolutional neural network architecture for text classification. Here, *y*_i_ is the label (RCT or not) for document *i*, **w** is a weight vector associated with the classification layer, and **x**_i_ is the vector representation of document *i* induced by the CNN

To represent a piece of text (a title and abstract), one stacks the embeddings of the constituent words, forming a matrix with dimensions equal to the length of the abstract (word count) by the length of the word embedding dimension (typically a few hundreds). The CNNs work by then passing *linear filters* parameterized by corresponding weight vectors over one or more adjacent word embeddings, starting at the beginning of the text and moving downward. In this way, each filter will produce a vector of scalar outputs of size proportional to the input text length. Filter outputs are then combined by performing *max‐pooling* on each filter output vector, ie, extracting the maximum value. Thus, each filter will ultimately generate a single scalar output. Finally, these are concatenated to form a vector representation of the entire abstract, which then becomes the input to a “softmax” classification layer, which predicts whether the study is or is not an RCT. Figure 5 depicts this schematically.

### 2.3 Data preprocessing, model features, and hyperparameter choices

For both SVMs and CNNs, we tokenized titles and abstracts into words and removed stopwords (common words with low informational content, eg, *this*, *and*, or *the*).

Each model type can be run with a very wide range of settings (known as *hyperparameters*), which can dramatically affect performance. These hyperparameters include not only unigrams through trigrams but also how much the model should be penalized for missing RCTs, and a large number of statistical parameters that affect model training (these are described in full in the Appendix).

For both SVMs and CNNs, we optimized associated model hyperparameters, evaluating performance on a withheld portion of the training data (20%). The single best‐performing set of hyperparameters for each model type at this stage was put forward for the final validation on the Hedges data. In all model types, we compensated for the class imbalance (see the section below) through both class weighting (adjusting the algorithm penalty for missing an RCT) and undersampling (altering the balance of the training set by random sampling so that it includes fewer non‐RCTs than RCTs).

Since the number of hyperparameter combinations is vast (we conservatively estimate at 3 billion unique possibilities for our CNN design), and each trial may take several hours to run, evaluating all possible combinations is not feasible. We therefore chose an approach known as Bayesian hyperparameter selection, which is the state‐of‐the‐art for this task.

For all models, we optimized hyperparameters using the *hyperopt* package using a *Tree of Parzen Estimators* algorithm with 500 iterations.^19^ For the final evaluations, we used the single best‐performing hyperparameters and trained on the entire Cochrane Crowd set. These best‐performing models were then evaluated on the Hedges dataset.

For SVMs, at the optimization stage, we compared unigrams, bigrams, and trigrams, each with and without the use of additional indicator features for words that appeared in the title. We evaluated the performance of raw token counts versus the use of *term frequency/inverse document frequency* weighting.^20^ We additionally optimized class weighting, the undersampling ratio, and L2 regularization strength.^21^ The optimal parameters chosen are shown in Box 2; the full details of the search space are provided in the Appendix.

Box 2 **Hyperparameters used for the final SVM**

Class weighting: RCTs: 12.4, non‐RCTs: 1.0

Sampling ratio: 9.2 non‐RCTs for each 1 RCT

Regularization type: L2

Regularization strength (alpha): 0.00092

Ngrams: 1

Number of iterations: 66

Loss type: Hinge

For CNNs, using the same process as the SVMs, we optimized the class weighting, the sampling ratio, and the L2 regularization strength. We additionally examined the effect of different numbers and sizes of filters (based on the ranges suggested by Zhang et al^7^) and differing ratios of dropout (where a proportion of units and connections are dropped at random during training; a widely used strategy to prevent overfitting in neural networks^22^). Finally, CNNs require a fixed vocabulary size; we examined the effect varying this size (where words were included in order of frequency in the training corpus). The final chosen hyperparameters are given in Box 3; the search spaces are provided in the Appendix.

Box 3 **Hyperparameters used for the final CNN**

Class weighting: RCTs: 3.86, non‐RCTs: 1.0

Sampling ratio: 6.0 non‐RCTs for each 1 RCT

Dropout: 0.160

Filter sizes: 1, 3, 5

Number of additional hidden layers: 0

Number of filters: 150

L2 normalization constant: 3.33

Maximum token features: 12500

Number of epochs: 2

#### 2.3.1. Handling class imbalance

There are far fewer RCTs than there are non‐RCTs. Such *class imbalance* can pose problems for standard learning algorithms, which typically aim to maximize overall predictive accuracy; in imbalanced scenarios, overall accuracy may be achieved by simply uniformly predicting the majority class. For both ML approaches, we used a *balanced sampling* technique, which aims to improve sensitivity in problems with class imbalance (noting that RCTs form a small minority of all articles).^23^, ^24^ In short, multiple training data sets are constructed from the available training data. Here, we construct such sets to include all RCT articles, but only a random subsample of the non‐RCTs (aiming to fully, or partially, balance the numbers of RCTs and non‐RCTs in the constructed set). To reduce the variance of model predictions and to make full use of the negative examples in the training data, this process is repeated to generate multiple models (25 in this analysis), and a final classification decided from the mean probability score across the models (known as *bagging* 25).

### 2.4 Ensembling

Ensembling is the strategy of using multiple ML models together, deriving a final decision through a voting scheme.^26^ Ensembles of models frequently perform better than their individual components. In addition to balanced sample bagging to compensate for class imbalance (described above), we make use of and evaluate ensembling in the following ways. First, we evaluate SVMs and CNNs both individually and as an ensemble. Second, to make use of the PubMed PT tag (which does not occur in the training data), we treat this tag as an additional binary classifier, so that the presence of the tag counts as a *vote* for the RCT class. The final ensemble score was the sum of normalized output scores ||
The scores produced by each model type are on different scales (CNNs producing probabilities between 0 and 1; SVMs producing non‐probability, unconstrained decision scores). We therefore used a simple normalization strategy based on the results from the training set: scores from each model type were *centralized* by subtracting the mean, and scales *standardized* by dividing by the standard deviation. from the component models. Scores were left as continuous for the primary analysis (areas under the receiver‐operating characteristics curves). Our secondary analysis comprised direct comparisons versus conventional database filters (being (1) PubMed PT and (2) Cochrane HSSS). In each case, we used a score cutoff that matched the performance of the comparison filter (matching the *specificity* of the PubMed PT and matching the *sensitivity* of the Cochrane HSSS).

### 2.5 Evaluation methods

Our primary outcomes were sensitivity and specificity at different threshold points, plotted as receiver operating characteristics (ROC) curves. Our evaluation dataset was the Clinical Hedges dataset. We reran the 2 principle control search filters (the Cochrane HSSS plus the PubMed PT tag) in January 2017, both to ensure the evaluation denominators matched exactly and also to make use of retrospective corrections of the PT tag, which would have taken place since the analysis of McKibbon et al.^9^

#### 2.5.1 Statistical analysis

Analyses were conducted in *R*, with area under the receiver operating characteristics (AUROC) curves and confidence intervals calculated using *pROC* package^27^; confidence intervals for differences in sensitivities and specificities versus controls were estimated using the modified Wald approach for differences in proportions with matched pairs, implemented in the *PropCIs* package.^28^ As a secondary measure, we present the *number needed to screen* (NNS) statistic.^29^ The NNS is analagous to the widely used Number Needed to Treat^30^ and is defined as the number of algorithm‐positive articles, which would need to be screened manually on average to retrieve one true RCT.

## 3 RESULTS

**Figure 6:**
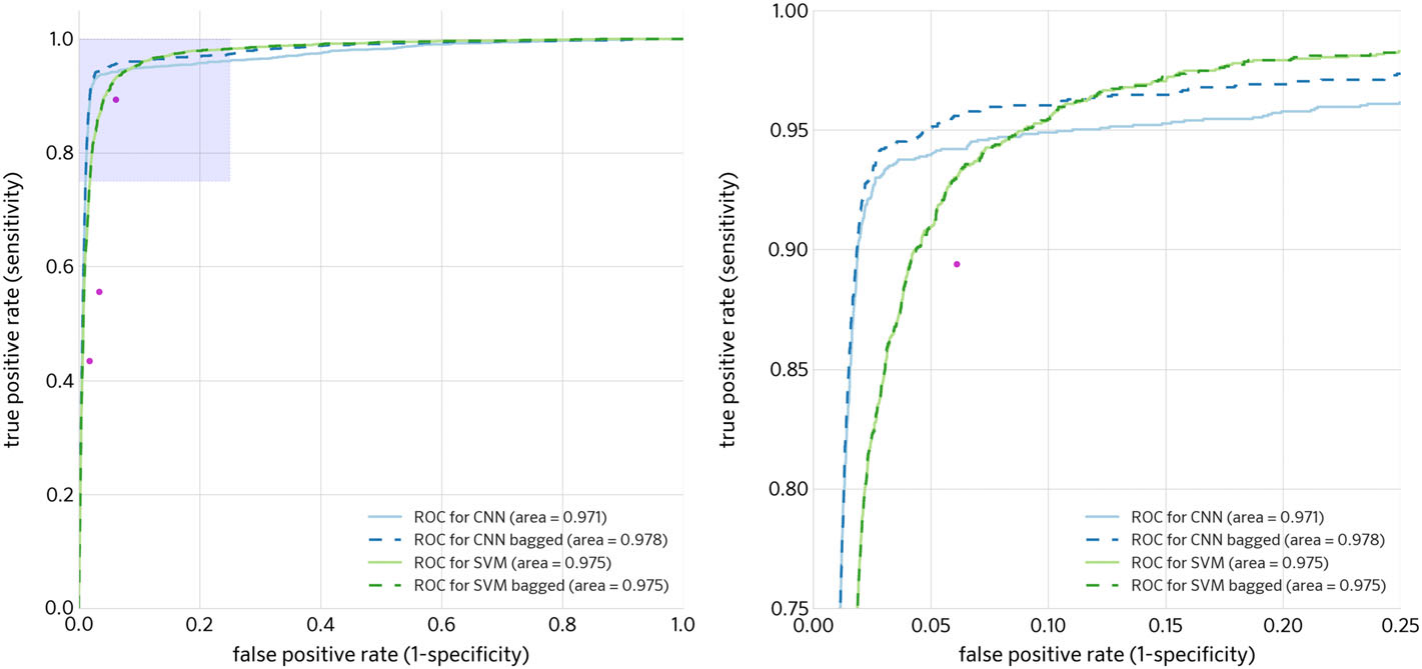
Receiver operating characteristics of the machine learning algorithms trained on plain text alone, (1) support vector machine, (2) convolutional neural network both single model, and *bagged* result of 10 models (each trained on all RCTs and a different random sample of non‐RCTs). The points depict the 3 conventional database filters, which use plain text only and do not require use of MeSH/PT tags. The blue shaded area in the left part of the figure is enlarged on the right‐side bottom section

**Figure 7:**
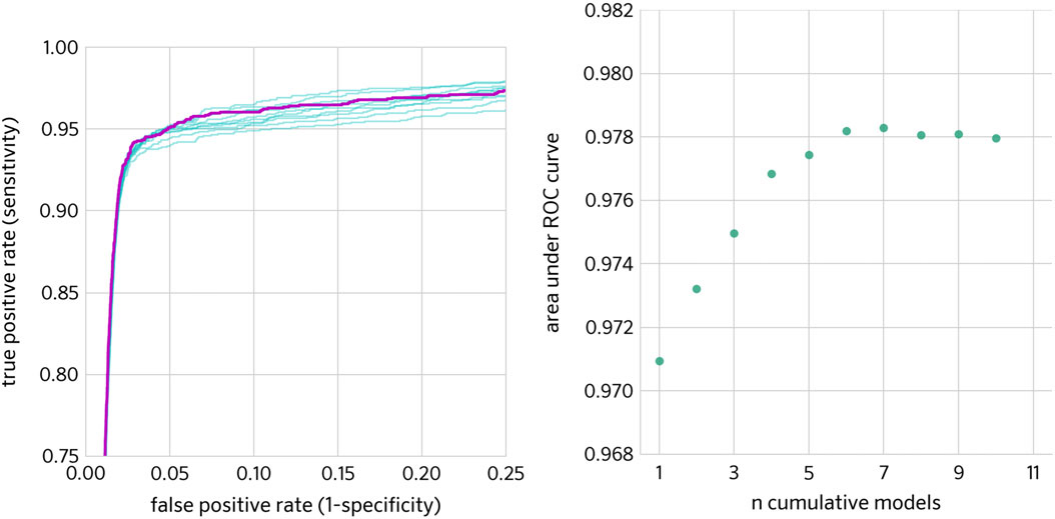
Left: Receiver operating characteristics curve (zoomed to accentuate variance); effects of balanced sampling: The individual models are depicted in light blue; the magenta curve depicts the performance of the consensus classification (the mean probability score of being an RCT from the component models). Right: Cumulative performance (area under receiver operating characteristics curve) of *bagging* multiple models trained on balanced samples. Performance increases until approximately 6 models are included, then is static afterwards

**Figure 8:**
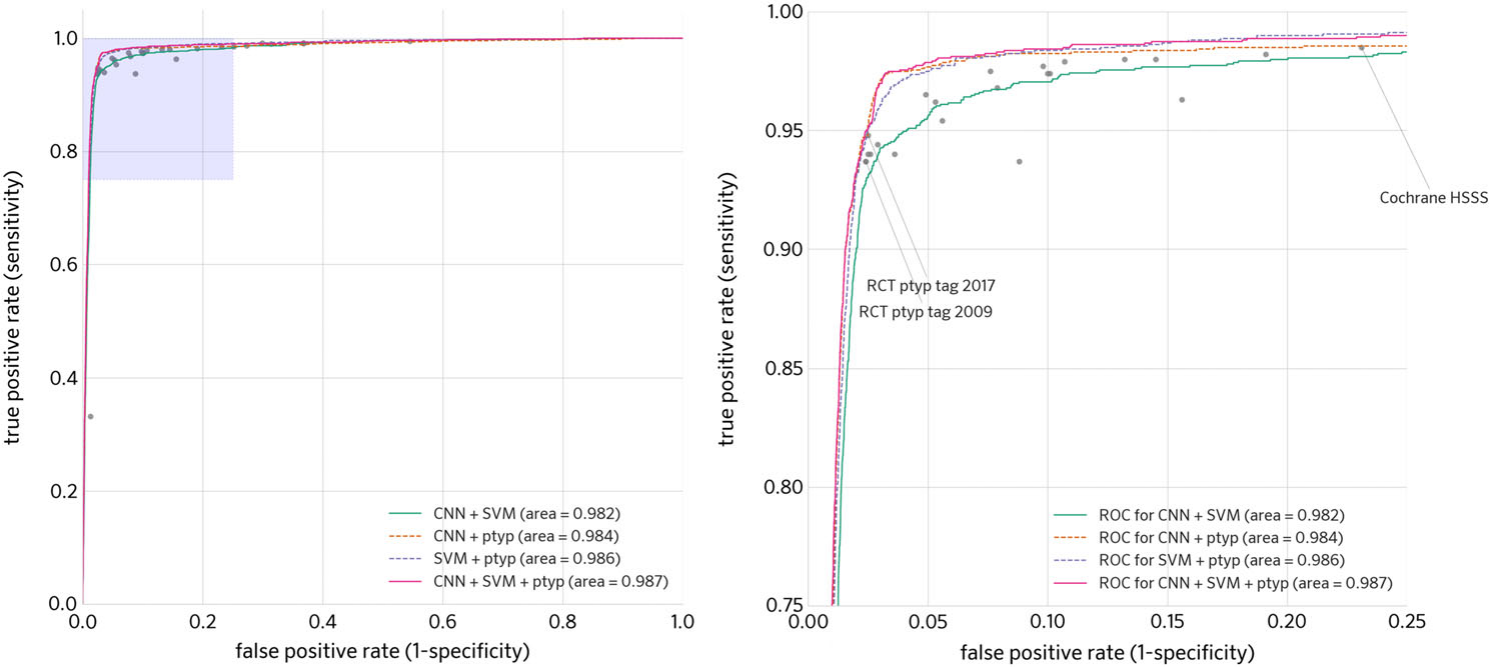
Receiver operating characteristics curve: Hybrid/ensembled models including use of the manually applied *PT* tag. The area bounded by the blue shaded area on the left‐hand plot is enlarged on the right to illustrate differences between models and conventional database filters. Note that the RCT PT tag has become more sensitive from 2009^9^ to 2017 (the reanalysis conducted here), reflecting the late application of the tag to missed RCTs including through data provided to PubMed by the Cochrane Collaboration^11^

Overall performance of the evaluated models on the Clinical Hedges dataset are presented in Table 1 (areas under the AUROC curves, with 95% confidence intervals) and in Figures 6, 7, 8.

**Table 1:** Area under receiver operating characteristics (ROC) curves for the ML strategies evaluated

Model	Area Under ROC Curve (95% CI)
SVM	0.975 (0.972‐0.979)
CNN	0.978 (0.974‐0.982)
SVM + CNN	0.982 (0.979‐0.985)
SVM + PT	0.987 (0.983‐0.989)
CNN + PT	0.984 (0.980‐0.988)
**SVM + CNN + PT**	**0.987 (0.984‐0.989)**

Bold text signifies best performing model.

Overall, the ensemble SVM + CNN + PT model discriminated best between RCTs and non‐RCTs (AUROC 0.987, 95% CI, 0.984‐0.989) The best‐performing model that did not require PT information was the SVM + CNN ensemble (AUROC 0.982, 95% CI, 0.979‐0.985).

Figure 6 shows ROC curves for the SVM and CNN models alone, alongside point estimates from the 3 competitor database filters (being those from McKibbon^9^ that did not require PT information). Visual inspection of ROC curves demonstrates subtly different characteristics of the CNN and SVM model: although both have high predictive performance. The CNN achieved better sensitivity at high specificity cutoffs (left part of curve), whereas the SVM achieved better sensitivity with lower specificity cutoffs (top part of curve).

The effects of sampling and bagging are shown in more detail in Figures 6 and 7. For the SVM model, bagging did not change the overall performance (Figure 6). For the CNN model, performance increased for each model added until 6 to 7 models; additional models added at this point did not improve performance (Figure 7).

Figure 8 shows the ROC curves for all of the ensemble strategies that incorporated PT information achieved greater accuracy than any of the database filters evaluated by McKibbon et al.^9^

### 3.1 Comparison with traditional filters

We present the sensitivity and specificity along with 95% confidence intervals of each evaluated model, using *high‐sensitivity* and *high‐precision* decision cutoffs in Tables 2 and 3.

**Table 2:** Performance on highly sensitive (systematic review) task, with comparison to conventional database filters

	Sensitivity^a^	Specificity	Precision	Number Needed to Screen
SVM	98.5 (97.8‐99.0)	71.7 (71.3‐72.1)	10.4 (9.9‐10.9)	9.6
CNN	98.5 (97.8‐99.0)	61.2 (60.7‐61.6)	7.8 (7.5‐8.2)	12.8
SVM + CNN	98.5 (97.8‐99.0)	68.8 (68.4‐69.3)	9.6 (9.1‐10.0)	10.4
**SVM + PT**	**98.5 (97.8‐99.0)**	**87.6 (87.3‐87.9)**	**21.0 (20.1‐22.0)**	**4.8**
CNN + PT	98.5 (97.8‐99.0)	82.1 (81.7‐82.4)	15.5 (14.8‐16.2)	6.5
SVM + CNN + PT	98.5 (97.8‐99.0)	84.0 (83.6‐84.3)	17.1 (16.3‐17.8)	5.8
Cochrane HSSS	98.5 (97.8‐99.0)	76.9 (76.6‐77.3)	12.5 (11.9‐13.1)	8.0

Abbreviation: Cochrane HSSS, the Cochrane Highly Sensitive Search Strategy.

a For the machine learning approaches, a predictive cutoff was chosen to achieve a fixed sensitivity of around 99%; better‐performing classifiers will achieve better specificity (ie, retrieve fewer non‐RCTs) at this sensitivity level. Bold text signifies best performing model.

**Table 3:** Performance on highly specific search task, with comparison to conventional database filters

	Sensitivity	Specificity^a^	Precision	Number needed to screen
SVM	82.3 (80.3‐84.1)	97.5 (97.4‐97.7)	52.5 (50.5‐54.5)	1.9
CNN	93.4 (92.0‐94.6)	97.5 (97.3‐97.6)	55.4 (53.5‐57.3)	1.8
SVM + CNN	93.3 (91.9‐94.4)	97.5 (97.4‐97.6)	55.6 (53.6‐57.5)	1.8
SVM + PT	95.1 (94.0‐96.2)	97.5 (97.3‐97.6)	55.8 (53.9‐57.7)	1.8
**CNN + PT**	**95.7 (94.5‐96.6)**	**97.5 (97.3‐97.6)**	**55.9 (54.0‐57.8)**	**1.8**
SVM + CNN + PT	95.1 (94.0‐96.2)	97.5 (97.3‐97.6)	55.8 (53.9‐57.7)	1.8
Pubmed *PT* tag as single search term	94.8 (93.6‐95.9)	97.5 (97.3‐97.6)	55.7 (53.8‐57.5)	1.8

Abbreviation: PubMed *PT*, *Randomized Controlled Trial* publication‐type tag as a single search term.

a For the machine learning approaches, a predictive cutoff was chosen to achieve a fixed specificity of 97.5%; better‐performing classifiers will achieve better sensitivity (ie, miss fewer RCTs) at this specificity level. Bold text signifies best performing model.

The evaluation of the high‐sensitivity strategies is shown in Table 1. The best‐performing model (the hybrid SVM model incorporating information from the *PT* tag [SVM + PT]) had significantly improved specificity compared with the Cochrane HSSS search filter: difference in specificity: 10.8% (95% CI, 10.5%‐11.2%), which corresponds to a precision of 21.0% versus 12.5%, and an NNS of 4.8 versus 8.0. In absolute terms, the SVM + PT model retrieved 5123 fewer of the 47 438 non‐RCTs in the Clinical Hedges set.

The best‐performing high‐precision ML strategy that did not require the *PT* tag (the SVM) had identical sensitivity, and 5.0% point inferior specificity to the Cochrane HSSS (95% CI, −5.4% to −4.5%). This corresponds to a reduced precision (10.4% versus 12.5%) and an NNS of 9.6 versus 8.0.

The evaluation of the highly specific strategies is shown in Table 2. The best‐performing model (the hybrid CNN model incorporating information from the PubMed *PT* tag) had a small increase in sensitivity compared with the Pubmed *PT* tag used alone; the difference was statistically significant (0.6%, 95% CI, 0.2%‐1.1%). In absolute terms, the best ML model retrieved an additional 10 of the 1587 RCTs from the Clinical Hedges dataset.

The best‐performing high‐precision ML strategy that did not require the *PT* tag (the CNN) had identical specificity, and 1.5% point inferior sensitivity to the *PT* tag alone. This corresponds to a reduced retrieval of 25/1587 RCTs.

## 4 DISCUSSION

We have presented an evaluation of RCT identification using state‐of‐the‐art ML approaches for text classification. Through analysis of ROC curves, we have shown that ML approaches outperform traditional database filters. For systematic reviews, ML leads to a substantial improvement in specificity compared to the Cochrane Highly Sensitive Search Strategy without harming sensitivity. For rapid reviews/high‐precision searching, ML has a (modestly) higher sensitivity than using the PubMed PT tag, without reducing specificity.

The ML‐based approaches to RCT identification have some appealing characteristics. First, where PT information is absent, they can be used with only the text from the title and abstract with only small reduction in performance. Second, the cutoff is very simply adjusted; particular needs of sensitivity versus specificity can be met from a single system. Third, given the ML system can predict probabilities, the articles may be ranked from highest to lowest probability (rather than a binary classification of being an RCT or not). For uses where a manual screening stage is still important, the researcher could screen a list of articles sorted from highest to lowest probability.^31^ The researcher may then make an informed decision to stop early based on either low likelihood that the remainder contains RCTs (for example in a conventional systematic review), or when resources (time or money) have been spent (for example in a rapid review).

### 4.1 Using the validated algorithms in practice

The results presented here suggest that an ensemble approach incorporating SVMs, CNNs, and PT information achieves the highest performance (measured as area under the ROC curve). The direct comparisons suggest that an SVM + PT ensemble may be best for high‐sensitivity search, and CNN + PT ensemble best for high‐precision searches. We found that under sampling non‐RCTs from the training data improved performance for all models; that bagging up to 6 to 7 CNN classifiers improved performance, but that there was no benefit for bagging SVMs.

To facilitate the use of ML in practice, we have made the algorithms and strategies evaluated above available as open‐source software. *RobotSearch* **
https://github.com/ijmarshall/robotsearch
 may be used as a substitute for traditional search filters. The user conducts their database search with the clinical terms using their preferred tool, but without using a traditional search filter. *RobotSearch* takes the search result (in RIS format) as input and generates a filtered list containing only articles classified as RCTs. By using widely accepted RIS format files, users can continue to use their usual bibliographic management tools for the other parts of their workflow.

We have additionally added the best‐performing high‐precision ML classifier to *RobotReviewer*, ††
https://robotreviewer.vortext.systems/ to verify whether uploaded articles are RCTs or not (and therefore whether they are eligible for further RCT‐specific processing, such as use of the Cochrane Risk of Bias tool). Currently, widely used versions of MEDLINE (eg, PubMed and Ovid) allow preset traditional filters to be applied at a click of a button. Ideally, ML approaches should be similarly easy to use: We encourage database producers to incorporate ML filters to facilitate this.

We have presented 2 common use cases to illustrate ML performance. However, any point may be chosen from the ROC curves depending on the use case—one key benefit of ML over database filters. For example, if a systematic reviewer required higher *sensitivity* than the Cochrane HSSS, a cutoff may be chosen to give 99.1% sensitivity and 77.2% specificity (compared with the 98.5% sensitivity, 76.9% specifity of the Cochrane HSSS: ML used with these parameters miss fewer RCTs and without harming later manual workload). We have provided a spreadsheet of the sensitivty and specificity of ML at various cutoff points as Supporting Information.

### 4.2 Strategies that include the publication‐type tag

**Figure 9:**
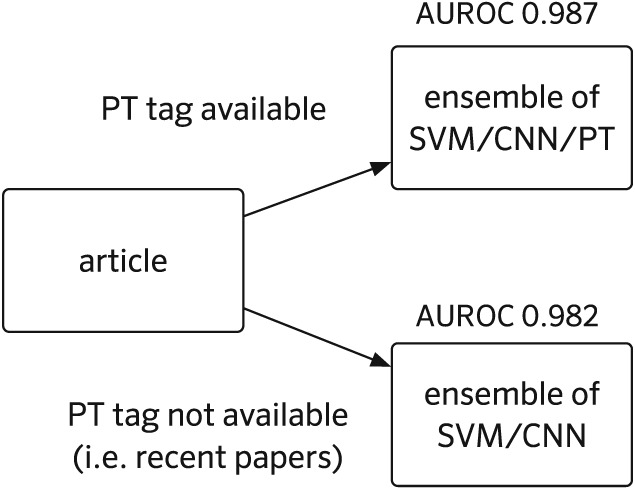
PubMed PT information is used where present; where not, the best‐performing text‐alone approach is automatically used, with a modest reduction in accuracy

Currently, the PT tag is applied manually by the PubMed team, although with a typical lag‐time of 250 days from publication. This is likely to shorten in future, as publishers begin to submit their own article classifications on publication (although this is likely to remain difficult for all but the largest journals, who have the resources to use indexing specialists). Therefore, both conventional filters and ML approaches that rely on this tag will be impaired for recent research. In instances where the PT tag is absent, conventional search filters using text alone are substantially impaired (Figures 2 and 6), whereas text‐alone ML approaches have only a modest reduction in performance (AUROC 0.987 with use of PT tag *versus* 0.982 without). In practice, the better‐performing ML models incorporating PT information can be used for most of the articles, with the text‐alone ML models used as a fallback where the information is absent. We have implemented this approach in our software (see Figure 9).

### 4.3 Strengths and weaknesses

One consideration is that the Clinical Hedges dataset comprises articles from a specific 2‐year period, which is now 15 years old. If there have been systematic changes in how trials have been reported (in titles and abstracts), this will affect the actual performance on trials conducted before and after this time period. A key pressure for change in reporting comes from the CONSORT guidelines, first published in 1996, which advise that RCT publications must include the term *Randomized Controlled Trial* in the title.^32^ Increased explicit reporting and standardized phrasing would be expected (in theory) to improve the performance of both ML and traditional database filters over time; conversely performance of all strategies may be lower in trials published earlier than the Hedges dataset.

## 5 CONCLUSIONS

We recommend that users of health research move towards using ML as a replacement for database search filters, since they have the potential to reduce workload and increase accuracy. Incorporating machine learning models, such as the ones presented here, into database search engines over time will make this process easier; in the meantime, we encourage the use of our open‐source software, which implements the approach validated here.

## FUNDING

This work is supported by the UK Medical Research Council (MRC), through its Skills Development Fellowship program (IM), grant MR/N015185/1; the National Institutes of Health (NIH) under the National Library of Medicine, grant R01‐LM012086‐01A1; “Semi‐Automating Data Extraction for Systematic Reviews”; Agency for Healthcare Research and Quality (AHRQ) grant R03‐HS025024 “Hybrid Approaches to Optimizing Evidence Synthesis via Machine Learning and Crowdsourcing”, and Cochrane, via Project Transform.

## AUTHOR CONTRIBUTIONS

All planned the paper. I.J.M. and B.C.W. coded the models. A.N.S. and J.T. developed the training data. I.J.M. and B.C.W. conducted the evaluation. All drafted, reviewed the manuscript, and signed off the final version.

## Supporting information

Data S1: Supporting Information

## HYPERPARAMETER SEARCH SPACES

### 1

**Table:**

Class weighting: RCTs: 1.0 to 30.0, non‐RCTs: 1.0 Sampling ratio: 0.2 to 10 non‐RCTs for each 1 RCT Dropout: 0.0 to 0.7 Filter sizes: [[1], [2], [3], [4], [5], [6], [7], [1, 3], [1, 3, 5], [3, 5, 7]] Number of hidden layers: [1, 2, 3] Hidden layer dimensions (if used): [50, 100, 150, 200, 250, 300, 350, 400] Number of filters: 50 to 250 L2 normalization constant: 0 to 5 Maximum token features: [1000, 5000, 10 000, 12 500, 15 000, 17 500, 20 000, 25 000, 30 000, 35 000] Number of epochs: Up to 50, with stopping patience of 1 for nonincreasing disciminance

Search space for CNN hyperparameters

**Table:**

Class weighting: RCTs: 1.0 to 30.0, non‐RCTs: 1.0 Sampling ratio: 0.2 to 10 non‐RCTs for each 1 RCT Regularization type: L2 Regularization strength (alpha): e^‐7 to − 1^ Ngrams: [1, 2, 3] Tf‐Idf weighting: [yes, no] Title indicator feature:[yes, no]* Number of iterations: 1 to 1000 Loss type: Hinge

Search space for SVM hyperparameters

* Separate n‐gram features were compiled for words appearing in the title and abstract. We hypothesized that certain words appearing in the title (for example, *randomized*) might have more importance than if they appeared in the abstract. A model that was able to weight these differently might therefore perform better.

## References

[1] Chalmers I , Enkin M , Keirse MJNC . Preparing and updating systematic reviews of randomized controlled trials of health care. Milbank Q. 1993;71(3):411‐437. 8413069

[2] Lefebvre C , Manheimer E , Glanville J . Searching for studies In: Higgins J, Green S, eds. Cochrane Handbook for Systematic Reviews of Interventions. 5.1.0 ed. The Cochrane Collaboration; 2011.

[3] Joachims T . Text categorization with support vector machines: learning with many relevant features In: Nédellec C, Rouveirol C, eds. Machine Learning: ECML‐98. Vol.1398 Lecture Notes in Computer Science Berlin, Heidelberg: Springer Berlin Heidelberg; 1998.

[4] McCallum, Andrew , Kamal Nigam , and Others . 1998 “A comparison of event models for naive Bayes text classification.” In AAAI‐98 Workshop on Learning for Text Categorization, 752:41–48 Citeseer

[5] Goldberg, Yoav . 2015 “A primer on neural network models for natural language processing.” arXiv [cs.CL] . arXiv.http://arxiv.org/abs/1510.00726.

[6] Kim, Yoon . 2014 “Convolutional neural networks for sentence classification.” Proceedings of the 2014 Conference on Empirical Methods in Natural Language Processing (EMNLP 2014), 1746–51.

[7] Zhang, Ye , and Byron Wallace . 2015 “A sensitivity analysis of (and practitioners' guide to) convolutional neural networks for sentence classification.” arXiv Preprint arXiv:1510.03820 , no. 1.http://arxiv.org/abs/1510.03820.

[8] Cohen AM , Smalheiser NR , McDonagh MS , et al. Automated confidence ranked classification of randomized controlled trial articles: an aid to evidence‐based medicine. J Am Med Inform Assoc: JAMIA. 2015;22(3):707‐717. 25656516 10.1093/jamia/ocu025 PMC4457112

[9] McKibbon KA , Wilczynski NL , Haynes RB . Retrieving randomized controlled trials from Medline: a comparison of 38 published search filters. Health Info Libr J. 2009;26(3):187‐202. 19712211 10.1111/j.1471-1842.2008.00827.x

[10] The Cochrane Library Oversight Committee . 2012 “Measuring the performance of the Cochrane library.” Cochrane Database Syst Rev 14 (11): ED000048. 10.1002/14651858.ED000048 PMC10846458 23235688

[11] Glanville JM , Lefebvre C , Miles JNV , Camosso‐Stefinovic J . How to identify randomized controlled trials in MEDLINE: ten years on. J Med Libr Assoc: JMLA. 2006;94(2):130‐136. 16636704 PMC1435857

[12] Wilczynski NL , Morgan D , Brian Haynes R . An overview of the design and methods for retrieving high‐quality studies for clinical care. BMC Med Inform Decis Mak. 2005;5(1):20 15969765 10.1186/1472-6947-5-20 PMC1183213

[13] The Cochrane Collaboration . 2015 “Central creation details.” The Cochrane Library .http://www.cochranelibrary.com/help/central‐creation‐details.html.

[14] Noel‐Storr, Anna . 2016 “The Embase Project.” ResearchGate .https://www.researchgate.net/project/The‐Embase‐project.

[15] Zhang, Ye , Iain Marshall , and Byron C. Wallace . 2016 “Rationale‐augmented convolutional neural networks for text classification.” arXiv [cs.CL] . arXiv.http://arxiv.org/abs/1605.\04469. 10.18653/v1/d16-1076 PMC5300751 28191551

[16] Mikolov, Tomas , Kai Chen , Greg Corrado , and Jeffrey Dean . 2013 “Distributed representations of words and phrases and their compositionality.” Advances in Neural Information Processing Systems 26 (NIPS 2013), 1–9.

[17] Pennington J , Socher R , Manning CD . Glove: global vectors for word representation. 2014;14:1532‐1543. EMNLP

[18] Pyysalo, Sampo , Filip Ginter , Hans Moen , Tapio Salakoski , and Sophia Ananiadou . 2013 “Distributional semantics resources for biomedical text processing.” Proceedings of Languages in Biology and Medicine .

[19] Bergstra, J. , Daniel L. K. Yamins , and D. D. Cox . 2013 “Making a science of model search: hyperparameter optimization in hundreds of dimensions for vision architectures.” *Proceedings of the 30th International Conference on Machine Learning*, 115–23.

[20] Salton G , Buckley C . Term‐weighting approaches in automatic text retrieval. Information Processing & Management. 1988;24(5):513‐523.

[21] Ng, A. Y. 2004 “Feature selection, L 1 vs. L 2 regularization, and rotational invariance.” Proceedings of the Twenty‐First International Conference …, 78.

[22] Srivastava N , Hinton G , Krizhevsky A , Sutskever I , Salakhutdinov R . Dropout: a simple way to prevent neural networks from overfitting. J Mach Learn Res: JMLR. 2014;15:1929‐1958.

[23] Wallace BC , Dahabreh IJ , Schmid CH , Lau J , Trikalinos TA . Modernizing the systematic review process to inform comparative effectiveness: tools and methods. J Comp Eff Res. 2013;2(3):273‐282. 24236626 10.2217/cer.13.17

[24] Wallace, Byron C. , Kevin Small , Carla E. Brodley , and Thomas a. Trikalinos . 2011 “Class imbalance, redux.” 2011 IEEE 11th International Conference on Data Mining, December. Ieee, 754–63.

[25] Breiman L . Bagging predictors. Mach Lear. 1996;24(2). Kluwer Academic Publishers‐Plenum Publishers):123‐140.

[26] Dietterich, Thomas G . 2000 “Ensemble methods in machine learning.” In Multiple Classifier Systems , 1–15 Lecture Notes in Computer Science 1857 Springer Berlin Heidelberg

[27] Robin X , Turck N , Hainard A , et al. pROC: an open‐source package for R and S+ to analyze and compare ROC curves. BMC Bioinformatics. 2011;12(March):77 21414208 10.1186/1471-2105-12-77 PMC3068975

[28] Scherer, Ralph . 2016 PropCIs package (version 0.2.5). https://cran.rstudio.com/web/packages/PropCIs/PropCIs.pdf.

[29] Rembold CM . Number needed to screen: development of a statistic for disease screening. BMJ. 1998;317(7154):307‐312. 9685274 10.1136/bmj.317.7154.307 PMC28622

[30] Barratt A , Wyer PC , Hatala R , et al. Tips for learners of evidence‐based medicine: 1. relative risk reduction, absolute risk reduction and number needed to treat. CMAJ: Can Med Assoc J = J de l'Assoc Medicale Canadienne. 2004;171(4):353‐358. 10.1503/cmaj.1021197 PMC509050 15313996

[31] Shemilt I , Khan N , Park S , Thomas J . Use of cost‐effectiveness analysis to compare the efficiency of study identification methods in systematic reviews. Systematic Reviews. 2016;5(1):140 27535658 10.1186/s13643-016-0315-4 PMC4989498

[32] Begg C , Cho M , Eastwood S , et al. Improving the quality of reporting of randomized controlled trials. The CONSORT statement. JAMA. 1996;276(D):637‐639. 8773637 10.1001/jama.276.8.637

